# Development of Taste Sensor to Detect Non-Charged Bitter Substances

**DOI:** 10.3390/s20123455

**Published:** 2020-06-18

**Authors:** Jumpei Yoshimatsu, Kiyoshi Toko, Yusuke Tahara, Misaki Ishida, Masaaki Habara, Hidekazu Ikezaki, Honami Kojima, Saeri Ikegami, Miyako Yoshida, Takahiro Uchida

**Affiliations:** 1 Graduate School of Information Science and Electrical Engineering, Kyushu University, 744 Motooka, Nishi-ku, Fukuoka 819–0395, Japan; yoshimatsu.jumpei.607@s.kyushu-u.ac.jp (J.Y.); ishida.misaki.812@s.kyushu-u.ac.jp (M.I.); 2 Institute for Advanced Study, Kyushu University, 744 Motooka, Nishi-ku, Fukuoka 819–0395, Japan; toko@ed.kyushu-u.ac.jp; 3 Research and Development Center for Five-Sense Devices, Kyushu University, 744 Motooka, Nishi-ku, Fukuoka 819–0395, Japan; 4 Intelligent Sensor Technology, Inc., 5–1–1 Onna, Atsugi-shi, Kanagawa 243-0032, Japan; habara.masaaki@insent.co.jp (M.H.); ikezaki.hidekazu@insent.co.jp (H.I.); 5 Faculty of Pharmaceutical Science, Mukogawa Women’s University, 11–68 Koshien 9-Bancho, Nishimiya, Hyogo 663-8179, Japan; h_kojima@mukogawa-u.ac.jp (H.K.); sae319@mukogawa-u.ac.jp (S.I.); miyakoy@mukogawa-u.ac.jp (M.Y.); takahiro@mukogawa-u.ac.jp (T.U.)

**Keywords:** taste sensor, lipid/polymer membrane, bitterness, non-charged substances, bitterness sensor, allostery

## Abstract

A taste sensor with lipid/polymer membranes is one of the devices that can evaluate taste objectively. However, the conventional taste sensor cannot measure non-charged bitter substances, such as caffeine contained in coffee, because the taste sensor uses the potentiometric measurement based mainly on change in surface electric charge density of the membrane. In this study, we aimed at the detection of typical non-charged bitter substances such as caffeine, theophylline and theobromine included in beverages and pharmaceutical products. The developed sensor is designed to detect the change in the membrane potential by using a kind of allosteric mechanism of breaking an intramolecular hydrogen bond between the carboxy group and hydroxy group of aromatic carboxylic acid (i.e., hydroxy-, dihydroxy-, and trihydroxybenzoic acids) when non-charged bitter substances are bound to the hydroxy group. As a result of surface modification by immersing the sensor electrode in a modification solution in which 2,6-dihydroxybenzoic acid was dissolved, it was confirmed that the sensor response increased with the concentration of caffeine as well as allied substances. The threshold and increase tendency were consistent with those of human senses. The detection mechanism is discussed by taking into account intramolecular and intermolecular hydrogen bonds, which cause allostery. These findings suggest that it is possible to evaluate bitterness caused by non-charged bitter substances objectively by using the taste sensor with allosteric mechanism.

## 1. Introduction

The taste felt by humans is comprised of five tastes (sweetness, umami, bitterness, sourness, and saltiness). These tastes are called “five basic tastes”, and each taste has an important role and characteristic for creatures to live. These “tastes” are subjective, because the way of feeling varies among people who taste it. Thus, an objective evaluation method of taste is in need.

For this reason, the technology that can quantify taste has been developed worldwide. Up to now, electronic tongues (e-tongues) and taste sensors are known as the devices which can qualify taste [1,2,3,4,5,6,7,8,9,10,11,12,13]. A taste sensor equipped with sensor electrodes, the surface of which is partially pasted with lipid/polymer membranes, measures taste electrically, and it has a characteristic of the “global selectivity” [1,2,3,4,5,6,7,8]. The sensor with this property does not discriminate each substance but discriminate and quantify each taste quality, as really found in the taste sense of humans. As a result, the taste sensor using lipid/polymer membranes can quantify the five basic tastes and astringent taste [1,2,5,6], and furthermore various kinds of foodstuffs such as coffee [14], tea [15], and beer [1,5], as well as pharmaceuticals [16,17]. It can also detect an inhibitory effect appearing in coexistent sweetness and bitterness [18] and a saltiness enhancement effect [19].

The concept of the taste sensor proposed by Toko was realized as the commercialized TS-5000Z taste sensing system. Each taste quality is detected selectively by the lipid/polymer membrane attached to the sensor electrode.

The sensors for bitterness of pharmaceutical products and bitterness of foods are called BT0 and C00, respectively. These sensors have high sensitivity and selectivity and can measure bitter substances. For example, BT0 can measure bitter substances of strong hydrophobic pharmaceutical products such as the quinine hydrochloride without responding to other taste qualities [1,2,5,6], and C00 can measure the acid bitter substances such as iso-α acid included in beer. However, the sensitivity of bitterness sensor BT0 and C00 for non-charged substances is too low, because the basic principle of the taste sensor is the membrane potential measurement mainly caused by change in the surface electric charge density accompanied by interactions with charged substances.

Caffeine is a white crystalline compound mainly included in tea leaves and coffee beans, and it is a kind of alkaloid belonging to purine. It is often used for beverages and pharmaceutical products, because it stimulates the central nervous system and serves to produce an awake action and excitement action after intake of caffeine [20]. Caffeine is an aromatic heterocycle organic compound comprised of a pyrimidine ring and an imidazole ring. Though alkaloids are usually basic nitrogen compounds, caffeine is non-charged molecule without basic properties. Our previous studies [21,22], where caffeine detection has been tried, revealed that the response to caffeine can be increased by immersing the membrane including the positively charged lipid into the solution of aromatic carboxylic acid (i.e., hydroxy-, dihydroxy-, and trihydroxybenzoic acids). Although this immersing process can be considered to modify the lipid/polymer membrane surface, the response mechanism was left unresolved. As a similar phenomenon, the sensitivity of a sweetness sensor for sugars (sucrose, trehalose, etc.) has been improved by making the surface modification [23,24,25].

In this study, non-charged bitter substances such as caffeine were measured using a surface modification method by immersing the sensor electrode in a modification solution which contains an aromatic carboxylic acid. As a result of surface modification, in which 2,6-dihydroxybenzoic acid (2,6-DHBA) was dissolved, it was confirmed that the sensor response increased with the concentration of caffeine, theophylline and theobromine. In addition, the threshold and increase tendency were consistent with those of human senses. Membranes modified with 2,6-DHBA or 2,4,6-trihydroxybenzoic acids (2,4,6-THBA) showed a remarkable response to caffeine. The moderate response appeared in the membranes modified by 2,3-DHBA and 2,5-DHBA, while no response was obtained in the case of 3,4-DHBA, benzoic acid, and 4-hydroxybenzoic acid (4-HBA).

When a hydroxy group in an aromatic carboxylic acid is adjacent to a carboxy group of the same molecule, they form an intramolecular hydrogen bond (H-bond) by letting the carboxy group dissociated [26,27]; that is, the -OH of the carboxy group easily releases H^+^ because the molecule tends to be stable. Therefore, when a membrane modified with an aromatic carboxylic acid is immersed in a caffeine sample, formation of the H-bond between the hydroxy group of aromatic carboxylic acid and the carbonyl group of caffeine breaks the intramolecular H-bond of the aromatic carboxylic acid, and the dissociated state becomes unstable. Then, it leads to the change in the membrane potential by taking back H^+^ from the solution. The interaction between one site (i.e., hydroxy group) and non-charged substances affects the dissociated state of another distant site (i.e., carboxy group). The binding of caffeine to one site of aromatic carboxylic acid causes the H^+^ binding on another distant site. In fact, two intramolecular H-bonds of aromatic carboxylic acid are formed in 2,6-DHBA and 2,4,6-THBA, one bond in 2,3-DHBA and 2,5-DHBA, while no bond is formed in 3,4-DHBA, benzoic acid, and 4-HBA; this number of intramolecular H-bonds agrees with the order of magnitude of response to caffeine as above. This is a new mechanism of potentiometric measurement utilizing allostery that occurs in many enzymes and receptors including taste receptors [28,29]. The allostery implies that binding with a ligand at one site affects an interaction with different or the same kind of ligand at another distant site of the receptor molecule. The present study revealed an allosteric mechanism that an aromatic carboxylic acid contained in the modified lipid/polymer membrane binds with non-charged bitter substances at the hydroxy group, which induces the H^+^ binding at the distant carboxy group. This allosteric mechanism will make it possible to develop a novel type of chemical sensor.

## 2. Experimental Section

### 2.1. Reagents

We purchased dioctyl phenyl-phosphonate (DOPP) and polyvinyl chloride (PVC) from FUJIFILM Wako Pure Chemical Corporation (Osaka, Japan), and tetradodecylammonium bromide (TDAB) from Sigma-Aldrich (USA). Caffeine, theophylline, and theobromine were purchased from Tokyo Chemical Industry Co., Ltd. (Tokyo, Japan). 2,6-Dihydroxybenzoic acid (2,6-DHBA), 4-hydroxybenzoic acid (4-HBA), 3,4-dihydroxybenzoic acid (3,4-DHBA), 2,3-dihydroxybenzoic acid (2,3-DHBA), 2,5-dihydroxybenzoic acid (2,5-DHBA), 2,4,6-trihydroxybenzoic acid (2,4,6-THBA), tannic acid, potassium chloride (KCl), quinine hydrochloride, tartaric acid, monosodium glutamate (MSG), and sucrose were obtained from Kanto Chemical Co., Inc. (Tokyo, Japan).

### 2.2. Lipid/Polymer Membrane

Before we make the measurement, we carried out a preconditioning process by immersing the sensor membrane in a reference solution (30 mM KCl and 0.3 mM tartaric acid) for 24–72 h. Our previous study proved that the electric response to caffeine increases conspicuously by soaking the lipid/polymer membrane containing positively charged lipids into the solution of aromatic carboxylic acid [21,22]. Thus, after cutting a lipid/polymer membrane and sticking it on a sensor electrode, it was immersed in 0.05 wt% of 2,6-DHBA or other aromatic carboxylic acid (benzoic acid, 4-HBA, 3,4-DHBA, 2,3-DHBA, 2,5-DHBA, and 2,4,6-THBA) solutions for 48 h. 

In this study, the lipid/polymer membrane was made by mixing 10 mL of 1 mM tetradodecylammonium bromide (TDAB) in tetrahydrofuran (THF) as a lipid, 1.5 mL dioctyl phenyl-phosphonate (DOPP) as a plasticizer, and 800 mg PVC as a polymer supporting reagent. Then, the mixture solution was poured into Petri laboratory dish (90 mm φ), and the membrane was formed because of volatilizing THF. The four sensor electrodes were used for the measurement of samples.

### 2.3. Measurement Procedure of Taste Sensor

**Figure 1:**
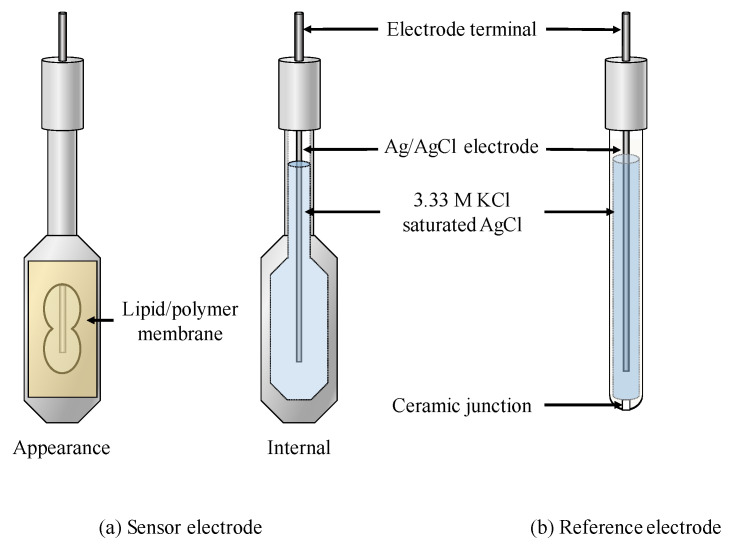
Sensor electrode (**a**) and reference electrode (**b**) of the taste sensor.

A commercialized TS-5000Z taste sensing system (Intelligent Sensor Technology, Inc., Kanagawa, Japan) was used for measurements. This device can be equipped with a reference electrode and at most eight sensor electrodes as the detection unit. In terms of sensor electrodes, a lipid/polymer membrane was stuck on the reception part of hollow sticks with a hole (Figure 1). The output of the sensor used the potential difference between the sensor electrode and the Ag/AgCl saturated KCl reference electrode. 

**Figure 2:**
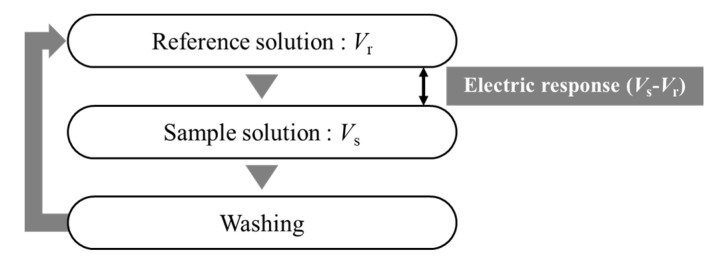
Measurement procedure of taste sensor.

Figure 2 shows the measurement procedure. At first, after the sensor electrodes were immersed in reference solution consisting of 30 mM KCl and 0.3 mM tartaric acid, the electric potential of the reference solution (*V*_r_) was measured for 30 s. Next, the electric potential of the sample solution (*V*_s_) was measured for 30 s, when the sensor electrodes were immersed in a sample solution. An electric response was calculated by the difference between *V*_r_ and *V*_s_,. i.e., vs. – *V*_r_. Finally, the membrane surface was refreshed by washing with the cleaning solution (10 mM KOH, 100 mM KCl, and 30 vol% EtOH).

The mean values and standard deviations (SDs) were calculated based on *n* = 4 (electrodes) × 4 (rotations) = 16 values of the electric responses in the same way as the previous paper [19].

### 2.4. Measurement of Caffeine and Allied Substances

We investigated electric responses of the membranes explained in Section 2.1 to caffeine and allied substances, theophylline and theobromine. These non-charged bitter substances are used for pharmaceutical products, and also included in food such as tea and coffee. The concentrations are shown in Table 1.

### 2.5. Sensory Test

The Weber–Fechner law says the magnitude of human sensory perception is in proportion to the logarithm of stimulus intensity [30]. We performed a sensory test of caffeine, theophylline, and theobromine. The evaluation method is shown as below.

Five well-trained panelists (healthy one male and four females: 35.8 ± 14.5 years old) evaluated the caffeine, theophylline, and theobromine samples using the previously described method [17]. The sample solutions had the concentration of 0.3, 1.0, 3.0, 10.0, and 30.0 mM of these three non-charged bitter substances. The panelists used quinine hydrochloride, the concentrations of which were 0.01, 0.03, 0.10, 0.30 and 1.0 mM, as standard bitterness solutions. The corresponding bitterness scores (τ) were 0, 1, 2, 3, and 4, in order. Prior to the test, the panelists included each 2 mL quinine hydrochloride solution in the mouth for 15 s and memorized the corresponding bitterness score. Then, the panelists assigned the bitterness score to each sample by including it in their mouth for 15 s. The ethical committee of Mukogawa Women’s University approved the experimental protocol of this study (No. 19–54) in advance, on 7 August 2019.

### 2.6. Confirmation of Sensor Selectivity 

The samples of five basic tastes and astringency taste were measured to confirm the selectivity of the lipid/polymer membrane used in this study. The compositions and concentrations of the measured samples which were measured are shown in Table 2 and the reference solution was used as a solvent. The membranes in the measurements were immersed to a preconditioning solution (i.e., modification solution) of 0.05 wt% of 2,6-DHBA solution for 48 h.

### 2.7. Influence of the Concentrations of Lipid and Modification Substance 

The concentrations of lipid TDAB and a modification substance 2,6-DHBA included in lipid/polymer membrane were changed as shown in Table 3. The other ingredients (DOPP and PVC) except the lipid TDAB were kept in the same as in Section 2.1. After cutting the membrane and sticking it on a sensor electrode, it was immersed 2,6-DHBA solution, as shown in Table 3, for 48 h. The 100 mM caffeine sample was measured using the above-formed membranes.

## 3. Results and Discussion

### 3.1. Measurement of Caffeine and Allied Substances

**Figure 3:**
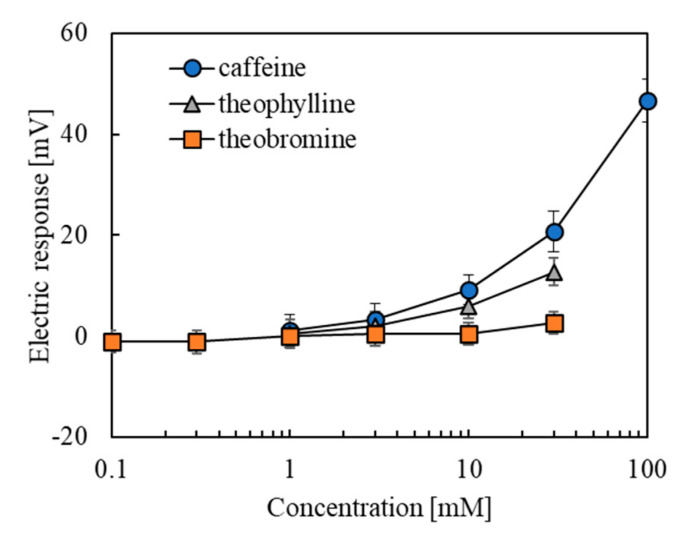
Responses of sensor with the lipid/polymer membrane to caffeine, theophylline, and theobromine.

The results of the measurement of caffeine and allied substances using the lipid/polymer membrane modified with 0.05 wt% 2,6-DHBA are shown in Figure 3. The error bar expresses the SD of the data of *n* = 4 (electrode) × 4 (rotation) = 16 values. The electric responses increased with the concentration of all the three samples. The response of caffeine sample increased to around 45 mV when the concentration was increased to 100 mM; caffeine showed the highest response in the measured three non-charged bitter substances. The response of theophylline increased to about 12 mV, slightly weaker than caffeine, at 30 mM theophylline. The response of theobromine increased to 2 mV at 30 mM; the response value was quite low, possibly due to the low water solubility of the theobromine (saturated at ~3 mM).

### 3.2. Sensory Test and Comparison with Taste Sensor Results

**Figure 4:**
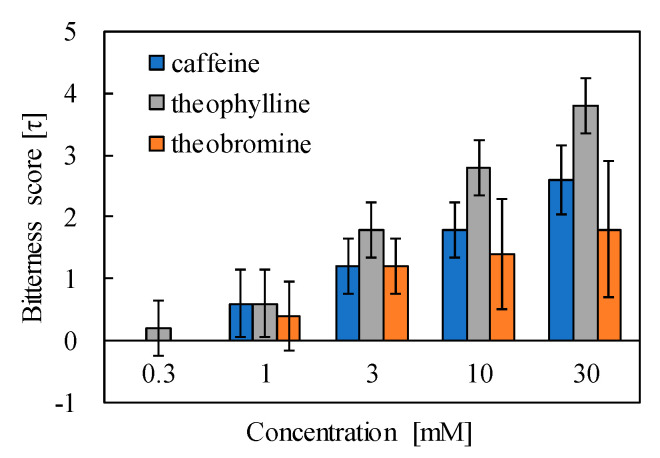
Result of sensory test for caffeine, theophylline, and theobromine.

The result of the sensory test is shown in Figure 4, which shows that the panelists began to feel the bitterness from around 1 mM and perceived the larger bitterness strength with the increasing concentrations. The bitterness scores of caffeine and theobromine at 0.3 mM showed zero.

**Figure 5:**
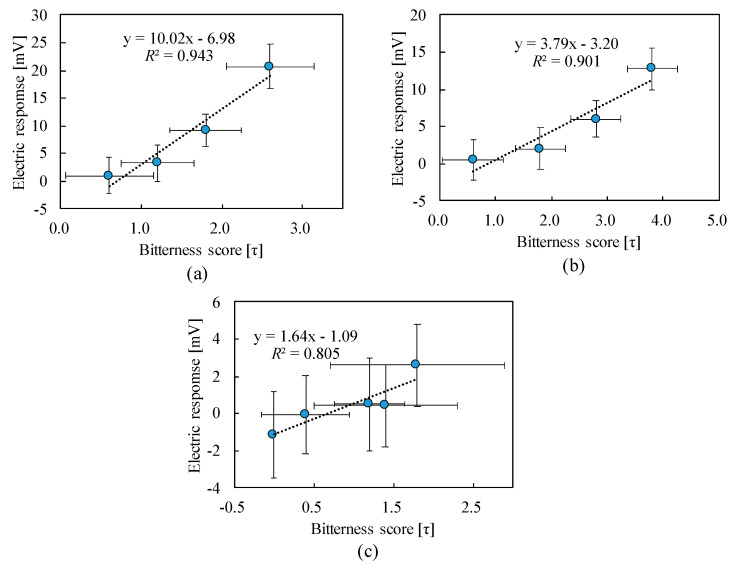
Relationships between the electric response of the taste sensor and bitterness score of the human senses: (**a**) caffeine, (**b**) theophylline, and (**c**) theobromine.

Figure 5 shows a relationship between the electric response of taste sensor and the bitterness level of the sensory test. The results of the electric response of taste sensor showed good correlations with the results of sensory test, as represented by *R*^2^ = 0.943, 0.901, and 0.805, respectively, for caffeine, theophylline, and theobromine. These results show that the electric response is in proportion to the bitterness intensity of samples, which reflects the logarithm of the concentration of each sample according to the Weber–Fechner law. In addition, the SDs of the electric responses were smaller than the sensory test, and therefore the evaluation using the taste sensor showed a superior performance than the human evaluation.

### 3.3. Confirmation of Sensor Selectivity

**Figure 6:**
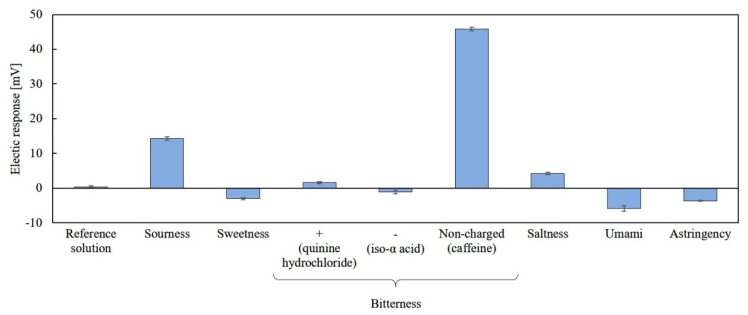
Response of taste sensor for five basic tastes and astringency.

Figure 6 shows the results of measurement for the five basic tastes and astringency samples using the lipid/polymer membrane modified with 0.05 wt% 2,6-DHBA. The sensor showed the highest response (about 45 mV) for caffeine, while it showed 14 mV to sourness with negligible responses (less than 5 mV) to other taste samples. Thus, the sensor electrode has a good sensitivity and selectivity to caffeine. Regarding the response to sourness, we can eliminate it in the sample containing caffeine and sour substances by using simultaneously the sourness sensor electrode which had been already commercialized, in a similar way to the case of coexisting sweetness and bitterness [18] or coexisting sweetness and saltiness [31].

### 3.4. Measurement of Caffeine by Surface Modification Method Using Various Aromatic Carboxylic Acids

**Figure 7:**
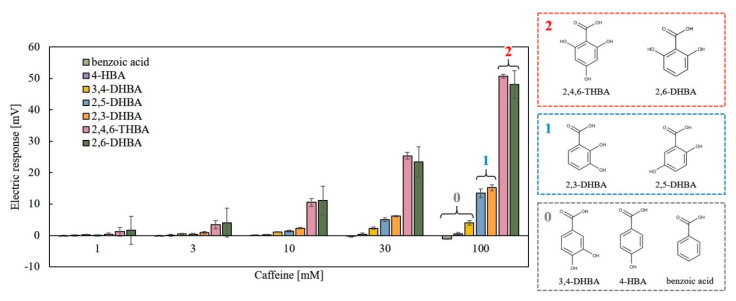
Responses of the membranes formed from the surface modification method using seven kinds of aromatic carboxylic acids to 100 mM caffeine. The numerical figures “2”, “1”, and “0” mean the number of intramolecular H-bonds.

Figure 7 shows the results of 100 mM caffeine measurement using lipid/polymer membranes modified with seven kinds of aromatic carboxylic acids. In particular, the membranes modified with 2,6-DHBA or 2,4,6-THBA showed a remarkable response to caffeine in accordance with a previous result [22]. The moderate response appeared in the membranes modified by 2,3-DHBA and 2,5-DHBA. No response was obtained for 3,4-DHBA, benzoic acid, and 4-HBA. These results can be interpreted by taking into account the property of aromatic carboxylic acids [26,27]: two intramolecular H-bonds of substances such as 2,6-DHBA and 2,4,6-THBA, which have two hydroxy groups at both ends of the carboxy group, are broken by caffeine binding with the hydroxy groups. As a result, the carboxy group takes back dissociated H^+^ from the solution, which results in the increasing surface electric charge density of the membrane and then increasing the membrane potential. Other chemical modification species, 2,3-DHBA and 2,5-DHBA, have one intramolecular H-bond, and therefore the change in surface electric charge density is smaller than the case of 2,6-DHBA and 2,4,6-THBA. As 3,4-DHBA, benzoic acid, and 4-HBA have no intramolecular H-bond, there appears to be no response to caffeine. Thus, a molecular structure having a carboxy group and a hydroxy group on both sides and undergoing intramolecular H-bond is effective for caffeine response.

### 3.5. Influence of the Concentrations of Lipid and Modification Substance

**Figure 8:**
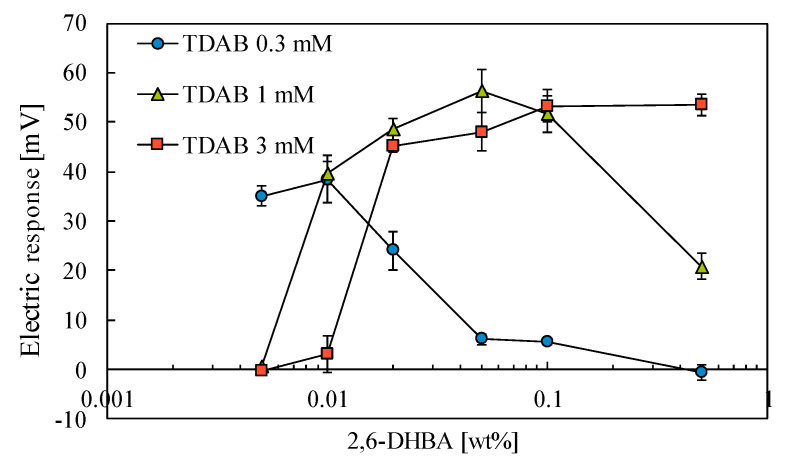
Change in electric responses for 100 mM caffeine with TDAB and 2,6-DHBA in different concentrations.

The electric responses to 100 mM caffeine are shown in Figure 8 as a function of the 2,6-DHBA concentration for three TDAB concentrations. The membrane containing 0.3 mM TDAB shows a peak in the electric response at the low 2,6-DHBA concentrations of 0.005–0.01 wt%. The membrane containing 1 mM TDAB has a peak at slightly higher 2,6-DHBA concentrations of 0.01–0.1 wt%. Furthermore, the membrane containing 3 mM TDAB shows a peak at the even higher 2,6-DHBA concentrations of 0.02–0.5 wt%. In other words, the membrane containing the tried middle concentration, 1 mM of TDAB, has a peak at the middle concentration area of 2,6-DHBA (0.01–0.1 wt%). These results suggest that the ratio of TDAB and 2,6-DHBA of the membrane constituents affects the response to caffeine. TDAB is positively charged, while 2,6-DHBA is negatively charged; therefore, it implies that these substances are needed to be included in the lipid/polymer membrane at the adequate ratio to get the high response to caffeine.

**Figure 9:**
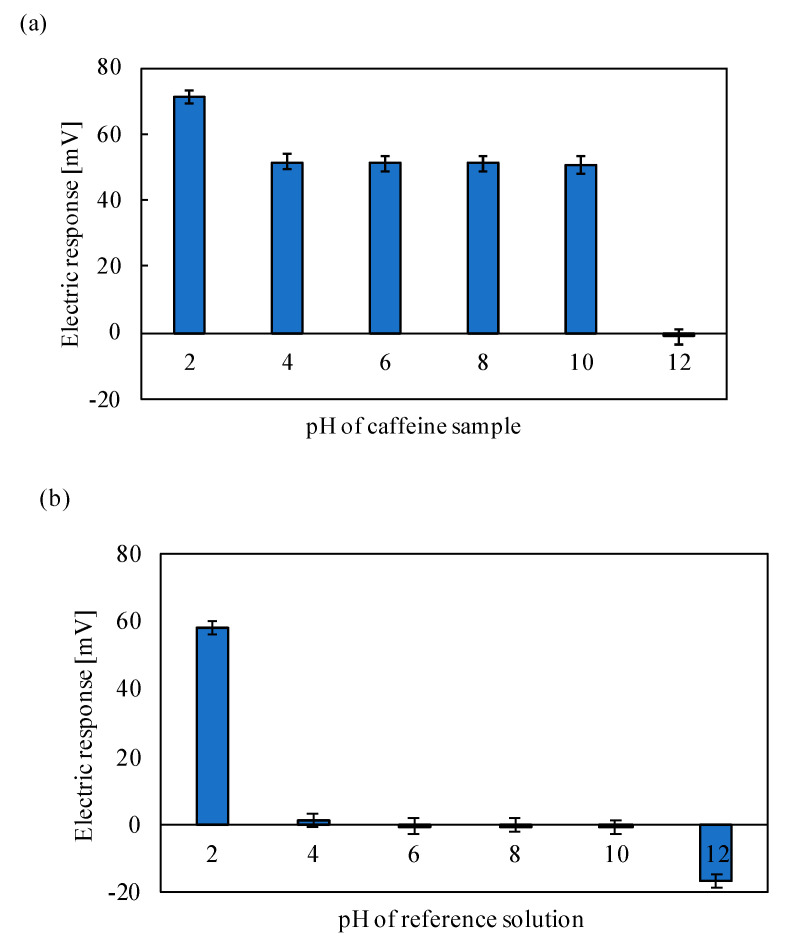
Electric potential response to 100 mM caffeine: (**a**) and the reference solution and (**b**) at different pHs from 2 to 12.

Figure 9 shows the electric potential response of the membrane with 1 mM TDAB and 1.5 mL DOPP treated with 0.05% wt% 2,6-DHBA solution, explained in Section 2.1, to 100 mM caffeine (a) and the reference solution (b) at different pH from 2 to 12. The origin of electric potential was taken at the reference solution at usual conditions, i.e., around pH 3.5 (see Figure 2). The responses at pH from 4 to 10 were the same as the results obtained in Figure 3, Figure 6 and Figure 8. The larger response appeared at pH 2, but no response appeared at pH 12. These two apparently opposite results at pH 2 and 12 can be explained as follows. Several carboxy groups of the aromatic carboxylic acids bind to H^+^ at extremely low pH such as pH 2, and therefore the surface charge density becomes more positive to result in more positive electric response than at or near neutral pH. As the response to the reference solution (b) is also at the same level as that to caffeine (b), the interaction of the membrane to caffeine occurs scarcely at this low pH. On the other hand, the situation at pH 12 is much different from those at lower pH from 2 to 10. At this extremely high pH, H^+^ dissociated from the carboxy group of an aromatic carboxylic acid cannot bind to the carboxy group; therefore, the membrane potential change does not occur at all. 

**Figure 10:**
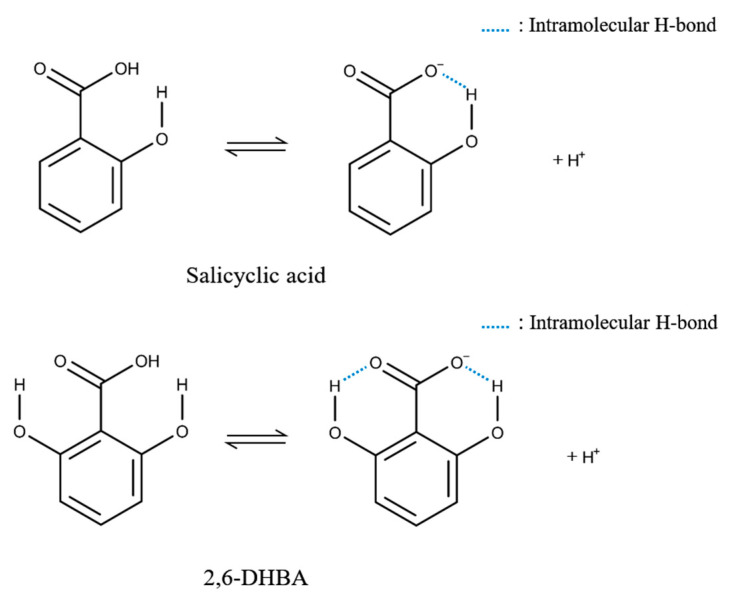
Intramolecular H-bond in salicylic acid and 2,6-DHBA.

The results shown in Figure 7, Figure 8 and Figure 9 can be summarized as follows. (1) The higher response to caffeine appears in the case of lipid/polymer membranes, the surface of which was modified with aromatic carboxylic acids with the ability to form the larger number of intramolecular H-bonds. (2) The ratio of positively charged TDAB and negatively charged 2,6-DHBA of the membrane constituents affects the response to caffeine. (3) The larger response appeared at pH 2, but no response appeared at pH 12. The acid dissociation constant (pKa) is lower when a hydroxy group is located at the ortho position, such as salicylic acid (2-HBA), than the case when a hydroxy group is located at the meta and para positions. This is due to formation of the intramolecular H-bond between the ortho-position hydroxy group and the adjacent carboxy group [26,27]. The carboxy group of salicylic acid tends to be stable by releasing H^+^, which implies low pKa (=2.97). According to pKa calculated by Marvin Sketch programs (ChemAxon Ltd.), another aromatic carboxylic acid, 2,6-DHBA, which has hydroxy groups on either side of the carboxy group, has an even lower pKa (=1.64) than salicylic acid because the two hydroxy groups each form an intramolecular H-bond with the carboxy group (Figure 10). X. Liao et al. reported that 2,3-, 2,4-, 2,5-, 3,4-, and 3,5-DHBA form cocrystal formers with piracetam, but 2,6-DHBA does not. They suggested two intramolecular H-bond formation between two hydroxy groups and carboxy group of 2,6-DHBA to result in no formation of cocrystal [32]. The pKa of benzoic acid, 4-HBA, 3,4-DHBA, 2,3-DHBA, 2,5-DHBA, 2.4,6-THBA are 4.08, 4.38, 4.16, 2.56, 2.53, and 1.95, respectively. These pKa values do not contradict with the result in Figure 7, i.e., showing the highest responses to caffeine in the case of 2,6-DHBA and 2,4,6-THBA with pKa lower than 2, the moderate response in 2,3-DHBA and 2,5-DHBA with pKa lower than 3, and the lowest responses in benzoic acid, 4-HBA and 3,4-DHBA with pKa higher than 4.

**Figure 11:**
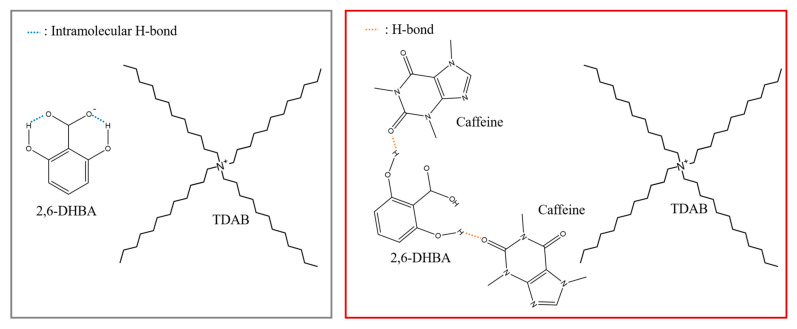
Mechanism of membrane electric potential increase (electric response) when caffeine is measured using the membrane whose surface is modified with 2,6-DHBA.

Based on this fact of pKa values and the obtained result of Figure 7, Figure 8, Figure 9 and Figure 10, we explain the response to caffeine by taking the case of 2,6-DHBA as an example. Two intramolecular H-bonds between the carboxy group and two hydroxy groups of 2,6-DHBA at the membrane surface are formed in the standard solution. When the sensor electrode is immersed in a caffeine sample, the carbonyl group of caffeine forms the H-bond with the hydroxy group of 2,6-DHBA as one of possible H-bonds [33]. Then, the already existing intramolecular H-bonds in 2,6-DHBA are broken, and thus the dissociated state becomes unstable. A 2,6-DHBA molecule becomes electrically neutral by taking back H^+^ from the solution. As a result, the membrane potential changes positively, as obtained in Figure 3, Figure 6, Figure 8 and Figure 10. As seen from Figure 11 illustrating this phenomenon, it is a kind of allostery, where the interaction that just occurred at one site affects the interaction at another distant site. The interaction between caffeine and hydroxy group of aromatic carboxylic acid affects the dissociated state of the carboxy group related to H^+^ binding. The binding of caffeine to one site of aromatic carboxylic acid causes the H^+^ binding on another distant site.

## 4. Conclusions

In this study, the measurement of non-charged bitter substances included in coffee was attempted using a taste sensor with a lipid/polymer membrane. The surface modification of the membrane was performed by immersing it in a modification solution containing an aromatic carboxylic acid (i.e., hydroxy-, dihydroxy- and trihydroxybenzoic acids) such as 2,6-dihydroxybenzoic acid. The comparison of the electric responses of the thus improved taste sensor to these substances with the sensory test showed a good correlation. The sensor electrode has good selectivity to non-charged bitter substances, because it did not respond to other basic five tastes or astringency except for sourness. The detection mechanism was discussed by taking into account intramolecular and intermolecular H-bonds, which cause allostery. It can be considered that the H-bond interaction between caffeine and hydroxy group of aromatic carboxylic acid affects the dissociated state of the carboxy group related to H^+^ binding. It is a kind of allosteric effect that the binding of caffeine to one site of aromatic carboxylic acid induces the H^+^ binding at another distant site. These findings suggest a possibility to objectively evaluate bitterness caused by non-charged bitter substances using the taste sensor with allosteric mechanism and also to develop novel chemical sensors utilizing a similar mechanism.

## Figures and Tables

**Table 1:** Concentrations of caffeine and allied substances.

Sample	Concentration
Caffeine	1, 3, 10, 30, 100 mM
Theophylline	1, 3, 10, 30 mM
Theobromine	0.1, 0.3, 1, 3, 10, 30 mM

**Table 2:** Compositions of five basic tastes and astringency sample solutions.

Taste	Composition	Concentration
Sourness	Tartaric acid	3 mM
Sweetness	Sucrose	1 M
Bitterness (+)	Quinine hydrochloride	0.1 mM
Bitterness (−)	Iso-α acid	0.01 vol%
Bitterness (non-charged)	Caffeine	100 mM
Saltiness	Potassium chloride	300 mM
Umami	Monosodium glutamate	10 mM
Astringency	Tannic acid	0.05 wt%

**Table 3:** Concentration of lipid, modification solution, and sample solution.

Composition	Concentration
TDAB	0.3, 1, 3 mM
2,6-DHBA solution	0.005, 0.01, 0.05, 0.1, 0.5 wt%
Caffeine solution	1, 3, 10, 30, 100 mM in reference solution
